# A Treatise on Nervous and Mental Diseases

**Published:** 1896-03

**Authors:** 


A Treatise on Nervous and Mental Diseases. By Landon Carter Gray, A. M., M. D. Second edition, revised and enlarged, with 172 illustrations and three colored plates. Octavo, pp. 733. Philadelphia: Lea Brothers & Co. 1895.

The first edition of this valuable book was reviewed in the March, 1893, number of the Journal, page 504, and was very carefully scrutinised and commented on. The new edition follows closely on the lines mapped out in the preceding, some additions having been made, the bibliographical references omitted, the glossary extended and the science of neurology brought down to the time of going to press. One fault with the first edition was the brevity of the chapters on mental diseases, also the entire omission of some chapters necessary for a complete understanding of psychiatry. These have been added, particularly the chapters on dementia, confusional insanity and delirium. An interesting chapter on the general principles of massage has been added, while the special applications are given under the separate diseases. No special notice is made of hydrotherapy, because, as the author remarks, he is not yet convinced that it is practical outside of the larger cities or that it has more value than mere cleanliness. Upon this point neurologists and hydrotherapeutists will hardly agree with the author. The therapeutical suggestions, although developed to their broadest in the first edition, are one of the features in this new edition, and nowhere in medical literature is the treatment of diseases more carefully considered than in this work. Not only are the medicinal and non-medicinal agents presented, but also those hygienic and dietetic measures which are often the physician's best reliance.

Many of the illustrations in the first edition were either faulty or imperfect. These have all been remedied, either reëxecuted or new ones substituted and the number considerably increased. The index is such a one as the busy practitioner delights to find, complete in every detail. Of the many excellent and worthy text-books on neurology published within the past five years, none are superior to Gray's and the physician makes no mistake in adding it to his medical library. W. C. K.

